# Proteome and Phosphoproteome Analysis in TNF Long Term-Exposed Primary Human Monocytes

**DOI:** 10.3390/ijms20051241

**Published:** 2019-03-12

**Authors:** Bastian Welz, Rolf Bikker, Johannes Junemann, Martin Christmann, Konstantin Neumann, Mareike Weber, Leonie Hoffmeister, Katharina Preuß, Andreas Pich, René Huber, Korbinian Brand

**Affiliations:** 1 Institute of Clinical Chemistry, Hannover Medical School, 30625 Hannover, Germany; welz.bastian@mh-hannover.de (B.W.); bikker.rolf@mh-hannover.de (R.B.); christmann.martin@mh-hannover.de (M.C.); neumann.konstantin@mh-hannover.de (K.N.); weber.mareike@mh-hannover.de (M.W.); leonie.hoffmeister@stud.mh-hannover.de (L.H.); preuss_katharina@gmx.de (K.P.); huber.rene@mh-hannover.de (R.H.); 2 Institute of Toxicology, Hannover Medical School, 30625 Hannover, Germany; johannes-junemann@gmx.de (J.J.); pich.andreas@mh-hannover.de (A.P.); 3 Core Unit Proteomics, Hannover Medical School, 30625 Hannover, Germany

**Keywords:** proteomics, phosphoproteomics, TNF long term exposure, monocytes, NF-κB

## Abstract

To better understand the inflammation-associated mechanisms modulating and terminating tumor necrosis factor (TNF-)induced signal transduction and the development of TNF tolerance, we analyzed both the proteome and the phosphoproteome in TNF long term-incubated (i.e., 48 h) primary human monocytes using liquid chromatography-mass spectrometry. Our analyses revealed the presence of a defined set of proteins characterized by reproducible changes in expression and phosphorylation patterns in long term TNF-treated samples. In total, 148 proteins and 569 phosphopeptides were significantly regulated (103 proteins increased, 45 proteins decreased; 377 peptides with increased and 192 peptides with decreased phosphorylation). A variety of these proteins are associated with the non-canonical nuclear factor κB (NF-κB) pathway (nuclear factor κB (NFKB) 2, v-rel reticuloendotheliosis viral oncogene homolog (REL) B, indolamin-2,3-dioxygenase (IDO), kynureninase (KYNU)) or involved in the negative regulation of the canonical NF-κB system. Within the phosphopeptides, binding motifs for specific kinases were identified. Glycogen synthase kinase (GSK) 3 proved to be a promising candidate, since it targets NF-κB inhibiting factors, such as CCAAT/enhancer binding protein (C/EBP) β. Our experiments demonstrate that both proteome and phosphoproteome analysis can be effectively applied to study protein/phosphorylation patterns of primary monocytes. These results provide new regulatory candidates and evidence for a complex network of specific but synergistically acting/cooperating mechanisms enabling the affected cells to resist sustained TNF exposure and resulting in the resolution of inflammation.

## 1. Introduction

After the acute phase of an inflammatory reaction of the immune system, a controlled and well-regulated resolution of the inflammatory process is required [1,2]. A dysregulation of the termination of inflammation can lead to an excess of the inflammatory reaction, the development of chronic inflammatory diseases, or even the inability of the organism to react to further pathogens, i.e., immune paralysis [2].

Different mechanisms participate in the coordinated termination of the inflammatory process, including the inhibition or controlled expression of pro- and anti-inflammatory cytokines and chemokines, the induction of specific receptor patterns, and the release of lipid mediators [3]. In addition, the development of different forms of tolerance induced by lipopolysaccharide (LPS) or tumor necrosis factor (TNF) [4,5], and a phenotype change of monocytes and macrophages or lymphocytes have been described [6]. TNF tolerance is characterized by genome-wide significant changes in gene expression patterns in monocytic cells, which influence a variety of cellular functions [7,8]. In this context, tolerance means that after preincubation with a specific stimulus, such as TNF, there is no response following restimulation [5]. In addition, several forms of cross-tolerance, e.g., between TNF and LPS, have been described [9,10]. It should be mentioned that the term tolerance is used because the expression of certain cytokines and chemokines is inhibited. However, tolerant cells can also display activated functionalities, which may be involved in the physiological termination of inflammation [5].

Both the inflammation-associated mechanisms modulating and terminating TNF-induced signal transduction after stimulation [11] and the mechanisms preventing the effects of re-stimulation (i.e., tolerance) are central themes in recent research [5]. To address these topics, we analyzed the proteome and phosphoproteome in TNF long term-incubated (48 h) primary human monocytes. Technically, this systemic analysis was performed using liquid chromatography and mass spectrometry (LC-MS/MS) with a data-dependent acquisition approach. The results of our experiments demonstrate that a combined proteome and phosphoproteome analysis of primary monocytes can be successfully applied yielding the identification of a defined set of proteins characterized by reproducible changes in expression and phosphorylation patterns. Among the significantly regulated proteins, several promising candidates were identified. Mechanistically, a variety of these proteins are associated with the non-canonical nuclear factor κB (NF-κB) pathway or involved in the negative regulation of the canonical NF-κB system and possess distinct binding motifs for kinases known for their contribution to the regulation of pro-inflammatory signaling, such as glycogen synthase kinase (GSK) 3. We conclude that our analyses provide promising candidates and indication for a balanced network of specific but synergistically interacting molecules, establishing resistance towards sustained TNF exposure and contributing to the resolution of inflammation.

## 2. Results

### 2.1. Protein Expression and Phosphorylation Patterns in Primary Human Monocytes Following Tumor Necrosis Factor (TNF) Long Term Treatment

**Figure 1:**
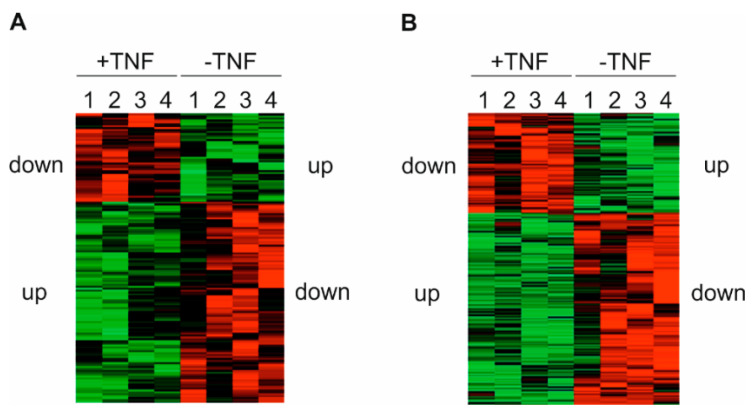
Protein expression and phosphorylation patterns in human monocytes following tumor necrosis factor (TNF) long term incubation. Primary human monocytes were incubated ± 400 U/mL TNF for 48 h (*n* = 4). Following Z-normalization of signal intensities among the 4 different liquid chromatography and mass spectrometry (LC-MS/MS) assays, a Perseus software-based bioinformatic clustering was performed. The heatmaps show increased (green) and decreased (red) expression of detected proteins (**A**) or phosphorylation of detected peptides (**B**) in respect of a statistically assumed value calculated on the base of the signal distribution.

To further elucidate the underlying mechanisms responsible for terminating TNF signaling and inducing TNF tolerance, we performed an LC-MS/MS-based analysis of both the proteome and the phosphoproteome using protein extracts of TNF incubated over long term (400 units (U)/mL TNF, 48 h), i.e., TNF tolerant primary human monocytes compared to naïve control cells (Supplement, Figure S1A). In 4 independent experiments (using monocytes from 2 individuals per experiment), 3180 proteins were identified in total following TNF long term incubation. Of these, 1772 proteins were upregulated and 1408 downregulated in comparison to untreated controls. In parallel, 1242 phosphorylated peptides were detected, of which 884 peptides showed increased and 358 decreased phosphorylation levels. Distinct and reproducible patterns of statistically significant changes in protein expression and phosphorylation were detected among all experiments (Figure 1A,B; Supplementary Figure S1B, Table S1, Table S2). In these experiments, the majority of proteins (i.e., two-thirds of each) displayed higher expression levels and showed an increase in the net amount of phosphorylated peptides following long term incubation with TNF. In addition, we also found that the expression and phosphorylation of a significant amount of proteins and peptides (approximately one-third of each) was reproducibly downregulated. Together, these data demonstrate that proteome-wide analyses of proteins and phosphopeptides via LC-MS/MS can be successfully applied to primary human monocytes.

### 2.2. Significantly Regulated Proteins and Phosphopeptides

**Figure 2:**
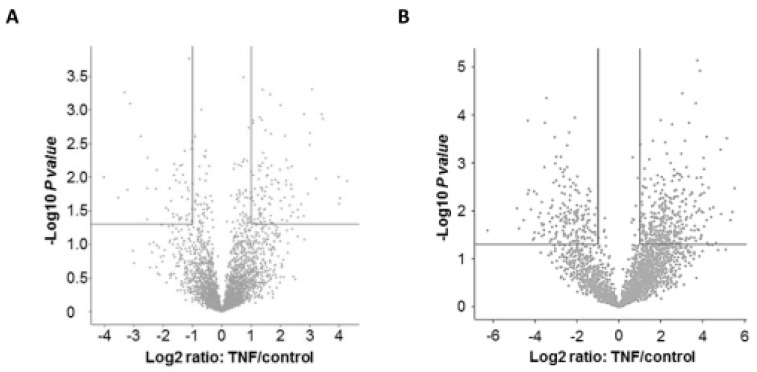

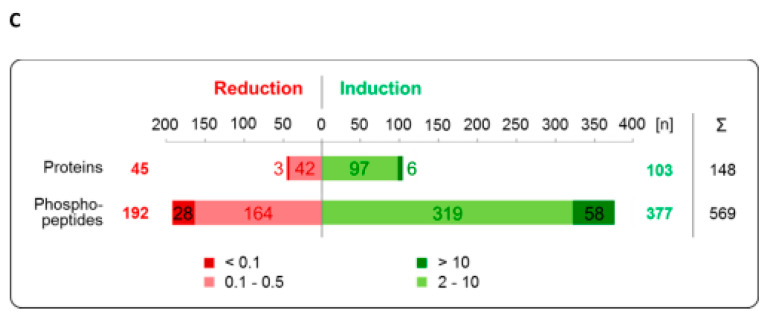
Significantly regulated proteins and phosphopeptides following TNF long-term incubation. The Volcano plots show a logarithmic representation of proteins (**A**) and phosphopeptides (**B**) identified in TNF long term-incubated cells in respect of the controls (protein TNF/protein control; phosphopeptide TNF/phosphopeptide control) as well as the subsets of significantly positively (upper right quadrant; cut off: 2-fold induction) and significantly negatively regulated (upper left quadrant, cut off: 0.5-fold induction) proteins and phosphopeptides (**C**). In TNF long term-incubated monocytes, 148 proteins (103 induced, 45 reduced) and 569 phosphopeptides (377 phosphopeptides induced, 192 phosphopeptides reduced) were significantly regulated.

In the next step the changes in the levels of total protein (protein TNF/protein control) were calculated, applying a cut off of ≥2.0- or ≤0.5-fold induction, respectively (Figure 2A,C). Using this strategy we found that the expression of 148 proteins significantly differed in TNF long term-treated cells, indicating that the expression of 103 proteins was increased (including 6 proteins > 10-fold), whereas 45 proteins were downregulated (3 proteins < 0.1-fold). In parallel, we determined the levels of phosphorylation (phosphopeptide TNF/phosphopeptide control) under this condition (Figure 2B,C). We identified 569 significantly regulated phosphopeptides—377 of which were more strongly phosphorylated than the controls (58 phosphopeptides > 10-fold), whereas 192 peptides showed a lower phosphorylation level (28 phosphopeptides <0.1-fold).

### 2.3. Validation of Proteome and Phosphoproteome Data by Western Blot Analysis

**Figure 3:**
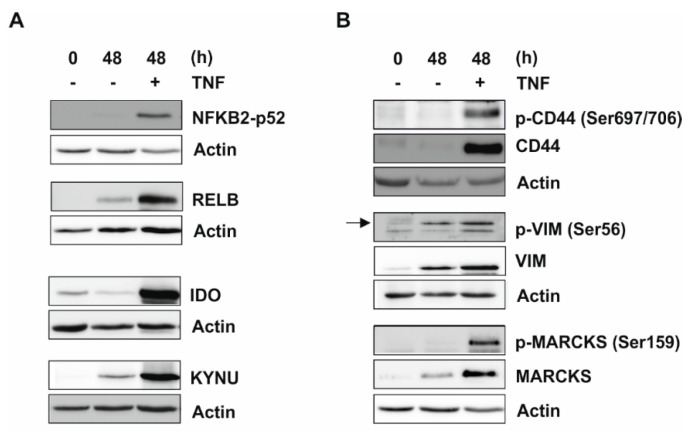
Validation of proteome and phosphoproteome results by Western blot analysis. Primary human monocytes were incubated ± 400 U/mL TNF for 48 h. In whole cell extracts, protein levels of p52, v-rel reticuloendotheliosis viral oncogene homolog (REL) B, indolamin-2,3-dioxygenase (IDO), and kynureninase (KYNU) (**A**), as well as (p-)cluster of differentiation (CD) 44, (p-)vimentin (VIM) (indicated by an arrow), and (p-)myristoylated alanine-rich C-kinase substrate (MARCKS) (**B**), which were identified by LC-MS/MS as increasingly expressed or phosphorylated following 48 h TNF treatment, were determined using Western blot analyses (*n* = 3; representative experiments). Loading control: Actin.

Proteome and phosphoproteome data were confirmed by Western blot analysis, selecting several proteins from these data sets (i.e., nuclear factor κB (NFKB) 2-p52, v-rel reticuloendotheliosis viral oncogene homolog (REL) B, indolamin-2,3-dioxygenase (IDO), and kynureninase (KYNU); Figure 3, Figure S2). To detect the phosphorylation of cluster of differentiation (CD) 44 and vimentin (VIM), we were able to utilize antibodies that were specific for the phospho-sites identified by our LC-MS/MS analysis. There is no phospho-antibody available to monitor the myristoylated alanine-rich C-kinase substrate (MARCKS) phosphorylation sites identified by LC-MS/MS analysis (i.e., Ser77 and Ser101). Therefore, a commercially available antibody against an alternative phospho-site (Ser159) was applied to analyze the phosphorylation of MARCKS under our conditions. Taken together, these analyses showed a considerable increase in proteins or phosphoproteins, respectively, which was consistent with the LC-MS/MS data.

### 2.4. Top Lists of the Proteome and the Phosphoproteome

Next, the top 25 proteins displaying higher expression levels in TNF long term-treated cells compared to naïve cells (protein TNF/protein control) were compiled (Table 1). Furthermore, Table 2 shows the top 25 peptides with significantly increased phosphorylation in TNF long term-treated monocytes. Functional bioinformatics analysis of these lists identified proteins that are involved in gene regulation, signaling, cellular structure, metabolism, and proteolytic processes, as indicated in the tables. This analysis of the proteome and the phosphoproteome provided interesting candidates for signaling termination and induction of TNF tolerance, as well as proteins regulating or influencing specific monocytic functions.

### 2.5. Expression and Phosphorylation of Proteins Associated with the Non-Canonical Nuclear Factor κB (NF-κB) Pathway or Involved in the Negative Regulation of NF-κB 

Our analyses revealed that numerous differentially expressed or phosphorylated proteins and peptides are involved in gene regulation and signaling. Table 3 shows a variety of proteins displaying increased proteome or phosphopeptide ratios of proteins associated with the non-canonical NF-κB pathway (i.e., NFKB2, RELB, IDO, KYNU), which contribute to the negative regulation of (canonical) NF-κB signaling [12,13]. We have previously shown that the dramatic suppression of the NF-κB system following TNF long term incubation is influenced by A20, cylindromatosis susceptibility gene (CYLD), IκB kinase (IKK), and receptor interacting protein (RIP) [8]. The controlled downregulation of NF-κB activation may be one of the key mechanisms to resolve signaling involved in inflammation. Our data suggest that a network of different proteins with either partially or completely differing functionalities and specific regulatory levels cooperate to terminate TNF-induced signaling and to orchestrate the state of TNF tolerance.

### 2.6. Increased Expression and Phosphorylation of p100/52, RELB, and p50

**Figure 4:**
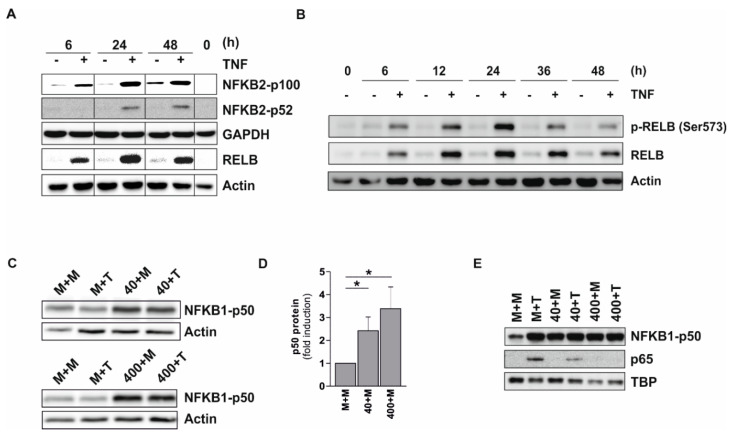
Increased expression of negative regulatory proteins of the NF-κB system. (**A**,**B**) Tohoku Hospital Pediatrics (THP-) 1 cells were incubated ± 400 U/mL TNF up to 48 h. (**A**) Protein levels of p100, p52, and RELB were determined in whole cell extracts at the indicated time points (representative experiment; *n* = 3); (**B**) Amount and phosphorylation of RELB were further assessed in a time course in cytosolic extracts (representative experiment; *n* = 3); (**C**–**E**) THP-1 cells were incubated ± 40 or 400 U/mL TNF for 48 h and then re-stimulated with 400 U/mL TNF (T) or incubated in medium (M) for 2 h. Subsequently, p50 protein levels were determined in whole cell extracts (**C**, representative experiment, *n* = 3) and analyzed using densitometry (**D**; mean ± standard deviation, *n* = 3). For calculation of p50 induction in pretreated but not re-stimulated cells, p50 expression in untreated cells was set as 1. (**E**) Protein levels of p50 and p65 were also detected in nuclear extracts (representative experiment; *n* = 3). Loading controls: Actin, glyceraldehyde-3-phosphate dehydrogenase (GAPDH), TATA-binding protein (TBP); * *p* ≤ 0.05.

The increase in NF-κB negative regulatory proteins was also monitored by time course experiments. Using Tohoku Hospital Pediatrics (THP-) 1 pre-monocytic cells incubated up to 48 h with 400 U/mL TNF, we observed a continuous increase in NFKB2-p100 accompanied by p52 formation, which stayed elevated up to 48 h (Figure 4A). A similar pattern was observed for RELB and phosphorylated (p-)RELB (Ser573) (Figure 4B), as well as NFKB1-p50 (Figure 4C,D). The amount of these proteins that could potentially inhibit transcriptional events in the nucleus was also monitored. Using p50 as an indicator, we observed a significant accumulation of this protein in the nucleus using both low and high TNF preincubation doses, whereas no such effect was observed for p65 (Figure 4E). In control cells, which were re-stimulated for 2 h, we also observed a strong increase in p65 in the nucleus, which was reduced when the low TNF long term incubation dose was used and completely abolished when the high dose was applied (Figure 4E). These data are in good agreement with observations previously published by our group [7]. This means, in cells tolerized with a low (but not a high) TNF dose, we still can detect a TNF-inducible increase of p65-containing complexes in the nucleus following short term restimulation. For further details concerning NF-κB-dependent signaling and its regulation, please see the information in the Discussion.

### 2.7. Identification of Glycogen Synthase Kinase (GSK) 3 Binding Motifs in Significantly Regulated Phosphopeptides and Functional Aspects

**Figure 5:**
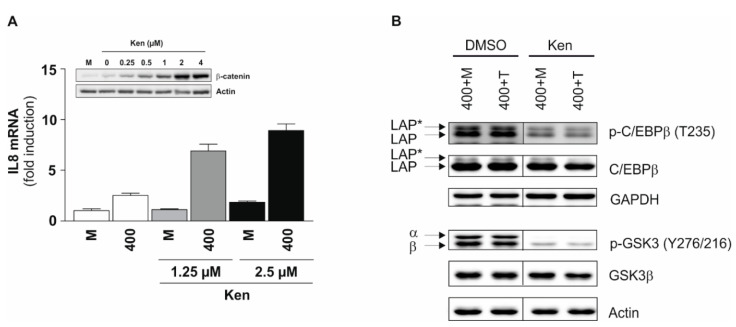
Involvement of GSK3 in TNF long term incubation. (**A**) Tohoku Hospital Pediatrics (THP-) 1 cells were incubated ± 400 U/mL TNF and glycogen synthase kinase (GSK) 3 inhibitor Kenpaullone (Ken; added 0.5 h before TNF treatment) for 48 h. The interleukin (IL) 8 mRNA amount was determined by qPCR (mean ± standard deviation, *n* = 3). The IL8 mRNA expression in untreated cells at 0 h was set as 1. The inset shows GSK3 inhibition-associated stabilization of β-catenin in unstimulated cells 48 h after addition of Kenpaullone to demonstrate its efficacy in the applied concentrations (representative experiment, *n* = 3; loading control: actin); (**B**) During long term incubation ± TNF (i.e., for 48 h), THP-1 cells were additionally treated ± 2.5 µM GSK3 inhibitor Kenpaullone (preincubation phase: 0.5 h). Following re-stimulation for 2 h, level and phosphorylation (Thr235) of C/EBPβ isoforms LAP* and LAP were determined in whole cell extracts. To demonstrate efficacy of Kenpaullone, GSK3α/β (auto-)phosphorylation at Tyr279/Tyr216 was determined (representative experiment, *n* = 3; loading controls: GAPDH, actin).

Next, we analyzed the kinase binding motifs of the phosphopeptides identified. A gene ontology-based cluster analysis revealed a prominent enrichment of binding motifs for several key immunoregulatory kinases, including GSK3α/β (Table 4). We have demonstrated before that GSK3 inhibition using GSK3 inhibitor SB216763 is able to reverse TNF tolerance [7]. Using an alternative GSK3 inhibitor, i.e., Kenpaullone, we also observed a dramatic increase in interleukin (IL) 8 mRNA expression following long term TNF incubation in a dose-dependent manner (Figure 5A). This indicates that GSK3 mediates inhibitory mechanisms during TNF long term incubation. In this case, IL8 was selected as major read-out parameter, since it is strongly inducible following short term TNF stimulation and prone to absolute TNF tolerance, i.e., downregulated following TNF long term incubation and not inducible following further restimulation [7]. Our data also showed an increase in the expression of CCAAT/enhancer binding protein (C/EBP) β (Table 3), which under certain conditions, exerts inhibitory effects on gene expression [14,15]. GSK3 has been suggested to be one of the kinases involved in modulating C/EBPβ DNA binding activity [16] and may exert a negative regulatory effect on the transactivation of specific genes [17,18]. A treatment with GSK3 inhibitor Kenpaullone within the TNF preincubation phase indeed resulted in a remarkable reduction of C/EBPβ phosphorylation at Thr235, as demonstrated for its isoforms, the liver-enriched activating proteins (LAP* and LAP; Figure 5B). Total LAP* and LAP protein expression remained constant. GSK3 inhibitor application concomitantly leads to the reduction of GSK3α/β phosphorylation at the activating sites Tyr279/Tyr216 (Figure 5B), which are prone to autophosphorylation [19]. In combination, these results support the hypothesis that GSK3α/β-mediated phosphorylation of C/EBPβ is involved in terminating TNF-induced gene expression during long term incubation. It has to be noted that our bioinformatics analyses also identified several other kinases that are potentially interesting candidates for mediating long term TNF response (Table 4).

## 3. Discussion

Following the acute phase of inflammation, a precise and highly controlled termination of this process is necessary [1,2,3] and a variety of potent mechanisms participate in its coordinated restriction. The regulatory network leading to termination of inflammation, however, has not been completely elucidated due to its enormous complexity. The understanding of the inflammation-associated mechanisms that modulate and switch off TNF-induced signal transduction after stimulation and understand mechanisms that prevent restimulation (tolerance) is a central topic of recent research [5,11]. Therefore, we analyzed the levels of proteins and phosphoproteins (represented by phosphopeptides) in TNF long term-incubated (48 h) primary human monocytes in a proteome-wide approach.

Our LC-MS/MS analysis yields distinct and consistent patterns in the respective heat maps of both the proteome and the phosphoproteome. These patterns of expression and phosphorylation demonstrate that in TNF-treated samples, approximately two-thirds of the significantly regulated proteins and peptides are characterized by an increased expression or phosphorylation, whereas the remaining one-third of the molecules show decreased levels. In absolute figures, the regulation of the phosphoproteome (377 increased and 192 decreased phosphoproteins, in total 569) is more extensive than the regulation of the proteome (103 increased and 45 decreased proteins, in total 148). Interestingly, the massive regulation of the phosphoproteome is even more pronounced with respect to the occurrence of highly regulated molecules, i.e., the phosphorylation of approximately 15% of the significantly regulated phosphoproteins was either strongly induced (˃10-fold induction; 58 of 377 phosphoproteins) or repressed (˂0.1-fold induction; 28 of 164 phosphoproteins), whereas only 6% of the proteins are regulated to this extent (6 of 103 increased and 3 of 45 decreased proteins). This suggests that posttranslational mechanisms provide a relevant contribution to the effects induced by long term TNF incubation. This is comparable with the results obtained in other biological contexts, such as differentiating blood cells [20]. It should also be noted that alterations in absolute protein levels may have a more significant influence on protein function than pure relative changes. Moreover, protein phosphorylation may exert a massive influence of protein levels, e.g., by directly influencing protein stability or indirectly regulating their expression levels by phosphorylated proteins. However, these aspects and interrelationships have not been addressed in our study due to specifications and limitations of the LC-MS analysis.

In our analyses, the identified patterns proved to be stable and reproducible, as reflected by comparable results among different experiments. Given the expected inter-individual variability in four approaches with pooled samples of two human individuals each, i.e., facing potential variations like those described in the context of skeletal muscle proteome analysis [21], this result is of particular importance. Thus, our results indicate a specific response of a defined set of monocytic genes and proteins to long term TNF incubation, an effect also enabling the distinct and reliable separation of treated and untreated samples via bioinformatics analysis of the collected protein and phosphopeptide data. From the technical point of view, our data collection also demonstrates that a reliable LC-MS/MS-based proteome and phosphoproteome analysis can be applied to primary cells. Most important, even primary human monocytes, i.e., a cell type generally regarded as difficult to culture, handle, and analyze, represent a suitable objective for corresponding experimental approaches.

Based on the proteome/phosphoproteome data, lists were created comprising candidate proteins and phosphoproteins with the most prominent increases in expression or phosphorylation levels following long term TNF incubation (i.e., top 25 of each). Functionally, when considering annotations given in the data bases UniProtKB (http://www.uniprot.org/) and GeneCards (http://www.genecards.org/), the differentially expressed proteins were especially involved in gene regulation (NFKB2, serine/arginine-rich splicing factor (SRSF) 2, RELB, SET nuclear proto-oncogene (SET), M-phase phosphoprotein (MPHOSPH) 8) and signaling (Sialic acid-binding immunoglobulin-type lectin (SIGLEC) 10, CD82, paired immunoglobin-like type 2 receptor α (PILRA), IL18), cell structure (SIGLEC10, myosin heavy chain (MYH) 11, ladinin (LAD) 1, CD82, apoptosis inhibitor of macrophage (AIM) 1, chloride intracellular channel (CLIC) 4, fascin actin-bundling protein (FSCN) 1, small acidic protein (SMAP), thyroid receptor-interacting protein (TRIP) 10), as well as metabolic processes (IDO1, KYNU, pyrophosphatase (PPA) 1, cytochrome P450 (CYP) 27A1, NAD(P)H Quinone Dehydrogenase (NQO) 1, arginine deiminase (ADI) 1, and adenylate kinase (AK) 4). Differentially phosphorylated proteins were predominantly associated with gene expression (eukaryotic translation initiation factor (EIF) 3G, RNA binding motif protein (RBM) 14, serine/arginine repetitive matrix protein (SRRM) 2, opioid growth factor receptor (OGFR), Fos-related antigen (FOSL) 2, RELB, La-related protein (LARP) 4B), signaling (Raftlin (RFTN) 1, TNF receptor-associated factor (TRAF) 1), and cell structure regulation (Integrin alpha (ITGA) 5, MARCKS, VIM, metadherin (MTDH), LAD1, CD44, nuclear mitotic apparatus protein (NUMA) 1, RFTN1, class II-associated invariant chain peptide (CLIP) 1, FK506 binding protein (FKBP) 15, protein tyrosine phosphatase receptor Type F interacting protein (PPFIA) 1).

**Figure 6:**
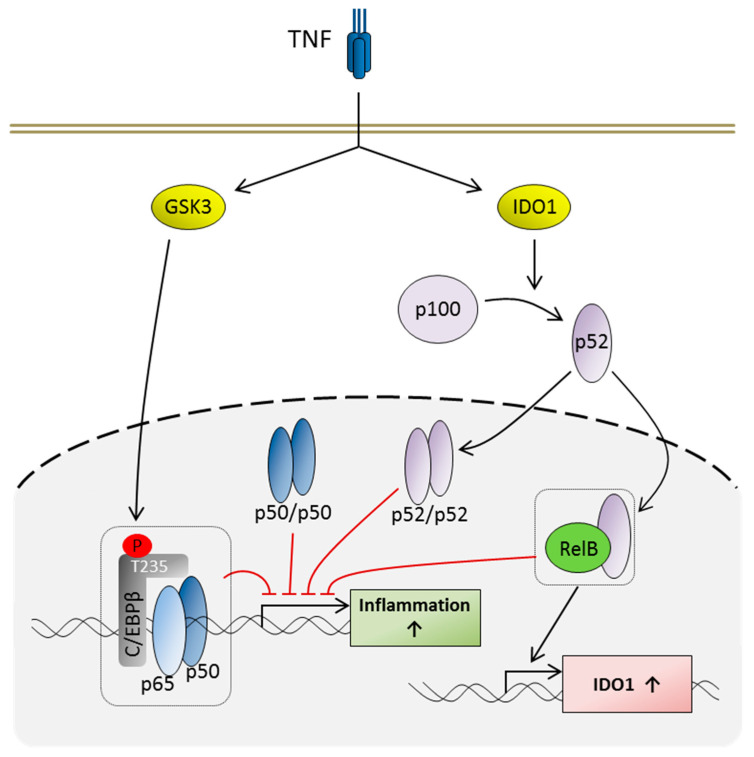
Inhibitory effects of GSK3, p52, RelB, and p50 on pro-inflammatory transcription following TNF long term exposure. TNF long term incubation leads to an activation of IDO1 and GSK3. NF-κB2-p100 is degraded to its active form p52 in an IDO-dependent manner. Following translation to the nucleus the non-canonical p52 and RelB homo- and heterodimers, as well as canonical p50, homodimers contribute to the inhibition of pro-inflammatory gene expression. RelB/p52 complexes are also involved in the induction of *IDO1* expression. GSK3 mediates phosphorylation of C/EBPβ (T235). Interaction of C/EBPβ with p65 inhibits the transcriptional activity of p65. Please find the relevant literature in the text of the Discussion.

Importantly, a remarkable number of all significantly regulated proteins and phosphoproteins (i.e., also including proteins not included in the top lists) are involved in the negative regulation of the NF-κB-dependent signaling (summarized in Table 3). For instance, our proteome analysis revealed an increased expression of members of the non-canonical NF-κB pathway [22], i.e., NFKB2 (p100/p52), an NF-κB subunit lacking transactivation domains, and its interaction partner RELB, a transactivating subunit which may, however, act as a repressor on certain promoters [12]. These data were confirmed by Western Blot showing a considerable elevation of p100 and RELB, as well as an increased RELB phosphorylation within 48 h in the presence of TNF. Similarly, the canonical NF-κB subunit p105/p50 (NFKB1; also transcriptionally inactive) exhibits increased levels in monocytic cells following long term TNF treatment in both the proteome analysis as well as our Western Blot data. In addition, TNF exposure leads to the translocation of p50 to the nucleus irrespective of the incubation time and the TNF dose used. This is consistent with previous results from our group showing a distinct induction of p50 over time [8], as well as an accumulation of p50 in the nucleus following TNF long-term incubation, and indicates a repression of canonical NF-κB-dependent gene transcription by transcriptionally inactive p50 homodimers. Taken together, our data suggest a shift towards non-canonical NF-κB-dependent signaling and a concomitant inhibition of the canonical NF-κB-dependent gene expression in long term TNF-treated cells (Figure 6).

Our proteome analyses also demonstrate a dramatic increase in IDO and KYNU protein concentrations. These enzymes, which participate in tryptophan metabolism [23] but also influence the inflammatory immune response [24], are connected to the non-canonical NF-κB pathway, since p52-RELB heterodimers are able to induce the expression of both IDO and transforming growth factor (TGF) β [25] (Figure 6). TGFβ, in turn, may induce IDO phosphorylation, which supports activation of IKKα, which is a kinase supporting the activation of the non-canonical pathway by inducing the formation of p52 from p100 [22] and Src homology region 2 domain-containing phosphatase (SHP-) 1, a protein tyrosine phosphatase promoting the activation of the non-canonical at the expanse of the canonical pathway due to the inhibition of Interleukin-1 receptor-associated kinase (IRAK) 1 [25,26]. In the present study, this may be reflected by the increased p100 level, which is accompanied by an increase in p52 at later time points (i.e., ≥24 h), indicating the generation of p52 from its precursor under these conditions. Moreover, the IDO-dependent metabolites kynurenine and kynurenic acid have been described to play a role in cytokine production (e.g., by increasing TGFβ expression or decreasing LPS-induced TNF secretion), immunomodulation, and the formation of LPS tolerance [24]. The KYNU-generated metabolites anthranilic acid and 3-hydroxyanthranilic acid also exhibit anti-inflammatory properties [27]. In the case of 3-hydroxyanthranilic acid, this effect appears to be associated with a suppression of NF-κB signaling via direct phosphoinositide-dependent kinase (PDK-) 1 inhibition [28].

Among the kinases potentially interacting with the phosphopeptides identified in our study (due to the presence of kinase binding sequences in their amino acid sequences), GSK3 was identified as a promising candidate. We and others have already suggested GSK3 as a key mediator of tolerance, since its inhibition using the GSK inhibitor SB216763 is able to reverse both TNF and TNF/LPS (cross) tolerance [7,10], though the detailed mechanisms remain elusive. Under the conditions applied in our studies, GSK3 appears to be predominantly activated via the reduction of inhibitory phosphorylation at Ser21 (GSK3α) and Ser9 (GSK3β) [7], since mRNA and protein levels, as well as the activating phosphorylation at Tyr279 and Tyr216 (which is also prone to auto-phosphorylation [19]), show no significant differences. GSK3 is known as a kinase targeting a variety of proteins negatively regulating the NF-κB system, also including candidates from Table 3, such as C/EBPβ, HSF-1, and NDRG1 [29]. Thus, GSK3 appears to mediate inhibitory mechanisms during TNF long term incubation. It is assumed that modulation of C/EBPβ DNA binding activity [16] and its negative regulatory effects on certain promoters are mediated by GSK3 [17,18]. Indeed, the application of the GSK3α/β inhibitor Kenpaullone results in a remarkable decrease in C/EBPβ phosphorylation at Thr235. Data from murine models suggest that phosphorylation at this site (i.e., Thr188 of murine C/EBPβ) is mediated GSK3-independently by mitogen-activated protein kinases, such as extracellular signal-regulated kinase (ERK), but acts as a prerequisite for further GSK3-dependent phosphorylation events at other C/EBPβ phosphorylation sites [17,18,30]. Other reports, however, indicate that GSK3 may play a role in Thr235/Thr188 phosphorylation, since GSK3 inhibition dramatically reduced fibroblast growth factor 2-induced C/EBPβ-Thr188 phosphorylation, suggesting that this site may represent a combined ERK-GSK3 consensus site [31]. In any case, it has already been demonstrated that Thr188 phosphorylation can lead to transcriptional repression of certain genes [18,31]. In the context of IL17-induced signal transduction, Thr188 phosphorylation and subsequent GSK3 activity appear to enable C/EBPβ to act as a mediator of suppressive signaling [18,32]. C/EBPβ has further been described to inhibit phosphorylation-dependent p65 activation by direct protein-protein interactions [15] and to induce the expression of A20 [33], a (de)ubiquitinase strictly controlling the transmission of TNF-dependent signaling [34]. In addition, C/EBPβ is an inhibitor of IκBα transcription [35], an effect which may contribute to the enhanced levels of p50 in the nucleus during TNF long term incubation. Thus, these data suggest the existence of a negative regulatory GSK3-C/EBPβ axis (Figure 6).

For HSF-1, it has been demonstrated that GSK3-mediated phosphorylation results in a decreased production of heat shock protein expression [29]. This allows the speculation that phosphorylated HSF-1 may suffer from a shift in its activity under these conditions, i.e., away from direct transcriptional activation towards indirect effects, such as the HSF-1-mediated suppression of NF-κB activation via the inhibition of p38 during stress reactions [36]. The regulatory effect of GSK3 on NDRG1 is still unknown [29], but it has been demonstrated that NDRG1 suppresses nuclear p65-p50-heterodimer translocation [37] and synergistically contributes to the repression of NF-κB-dependent gene expression by stabilizing IκBα, an effect mediated by NDRG1-dependent downregulation of IKKβ expression [38]. Taken together, the identification of an enrichment of GSK3 binding motifs in significantly regulated phosphoproteins (including NF-κB inhibiting factors) further supports the assumption that GSK3 plays a decisive role in the regulatory processes governing the termination of inflammation and TNF tolerance.

Other proteins from our list further contribute to the inhibition of NF-κB-dependent signaling (including promyelocytic leukemia protein (PML), decapping mRNA protein (DCP) 1A, nuclear receptor co-repressor (NCOR) 1 and 2, sirtuin (SIRT) 1, tumor suppressor p53-binding protein (TP53BP) 1, SIGLEC10, calpastatin (CAST), TRAF1, TRAF-type zinc finger domain-containing protein (TRAFD) 1, tumor necrosis factor α-induced protein (TNFAIP) 2), which, taken together, suggests that a whole arsenal of proteins is expressed or phosphorylated to inhibit (NF-κB-dependent) pro-inflammatory signaling at several regulatory levels and may represent functional key aspects during the termination of inflammatory processes and the development of TNF tolerance.

Our data reveal that reliable LC-MS/MS-based analyses of both the proteome and the phosphoproteome can be successfully applied to primary human monocytes, yielding distinct and reproducible patterns of differentially expressed and phosphorylated genes. Moreover, our results provide both new promising candidate genes and an indication for a complex network of specific but synergistically acting and cooperating mechanisms occurring during the termination of pro-inflammatory signaling and the formation of tolerance, thus enabling the affected cells to resist the continuing presence of high TNF concentrations and to resolve inflammation.

## 4. Material and Methods

### 4.1. Isolation of Primary Human Monocytes and Cell Culture

Freshly obtained blood samples (500 mL, heparinized) from healthy donors were provided by the Institute of Transfusion Medicine, Hannover Medical School. Informed patient consent was obtained, and the experiments were approved by the Hannover Medical School ethics committee in accordance with the Declaration of Helsinki (No. 388-2008, 02 December 2008). Monocytes were isolated as previously described [7] using the Monocyte Isolation Kit II (Miltenyi Biotec, Bergisch Gladbach, Germany) for negative cell selection according to manufacturer’s instructions. Purity of isolated monocytes (>90%) was assessed by flow cytometry. Primary human monocytes, human monocytic THP-1 cells, and HeLa cells (Deutsche Sammlung von Mikroorganismen und Zellkulturen, Braunschweig, Germany) were cultivated at 37 °C with 5% CO_2_ and 95% humidity in RPMI 1640 medium supplemented with 7.5% fetal calf serum (FCS), 100 U/mL penicillin, and 100 mg/mL streptomycin (Biochrom, Berlin, Germany). Medium for primary monocytes was additionally supplemented with 2% oxaloacetate/pyruvate/insulin media supplement (Sigma-Aldrich, St. Louis, MO, USA) and 1% minimum essential medium non-essential amino acids solution (Thermo Fisher, Bonn, Germany). Primary human monocytes and THP-1 cells were cultured in 2 mL medium in 12-well plates at a density of 4–5 × 10^6^ and 5 × 10^5^ cells/well, respectively. HeLa were plated at 2 × 10^5^ cells/well in 6-well plates (Sarstedt, Nümbrecht, Germany) containing 2 mL medium [39].

### 4.2. Reagents

TNF was purchased from Sigma-Aldrich (Darmstadt, Germany) and PMSF (a protease inhibitor applied for validation experiments) from Roth (Karlsruhe, Germany). Inhibitor experiments were performed using the GSK3α/β inhibitor Kenpaullone (Sigma-Aldrich). For Western blotting, antibodies specific for CD44 (156-3C11), p-C/EBPβ (Thr235), GSK3β (27C10), IDO (D5J4E), MARCKS (D88D11), NFKB1-p105/p50, NFKB2-p100/p52, p65 (L8F6), RELB (C1E4), VIM (5G3F10), p-VIM (Ser56; all from Cell Signalling, Danvers, MA, USA), p-CD44 (Ser706; Santa Cruz Biotechnology, Santa Cruz, CA, USA), C/EBPβ (E299; Abcam, Cambridge, UK), KYNU (Biozol, Eching, Germany), p-MARCKS (Ser159), p-RELB (Ser573; Thermo Fisher), p-GSK3α/β (Tyr279/Tyr216), actin (11C), GAPDH, and TBP (Sigma-Aldrich) were used. Horseradish peroxidase-coupled secondary antibodies were purchased from Cell Signalling or Vector Laboratories (Burlingame, CA, USA). All media and reagents were of the best available grade and routinely tested for endotoxins with the Limulus Amoebocyte Lysate assay (Lonza, Basel, Switzerland).

### 4.3. Protein Digestion and Fractionation by Strong Cation Exchange (SCX)

For LC-MS-based analyses, primary human monocytes incubated for 48 h ± 400 U/mL TNF were harvested by centrifugation (5 min, 400× *g*, 4 °C). Following a washing step using phosphate-buffered saline (4 °C), cells were lysed using buffer P (8 M urea, 4% 3-[(3-Cholamidopropyl)-dimethylammonio]-propan-sulfonate (Sigma-Aldrich), 30 nM Tris, 1% Nonidet P-40 (AppliChem, Darmstadt, Germany), 1% HALT^TM^ phosphatase inhibitor (Thermo), and EDTA-free cOmplete^TM^ protease inhibitor (Sigma-Aldrich) cocktails). To gain sufficient amounts of proteins, cell lysates of monocytes derived from 2 independent individuals were pooled for subsequent LC-MS analyses. 

To prepare for LC-MS analyses, samples were treated as described earlier [40]. Briefly, proteins were reduced with dithiothreitol (DTT; 5 mM) for 1 h at 37 °C and alkylated with iodoacetic acid (10 mM) in the dark at room temperature for 30 min. DTT was added at a final concentration of 5 mM to stop alkylation. Lysates were diluted with 50 mM ammonium bicarbonate to a final urea concentration below 4 M and proteins were digested with Lys-C (Fujifilm Wako, Neuss, Germany) at a 1:150 enzyme/protein ratio for 4 h at 37 °C, and subsequently with trypsin (1:80; Promega, Mannheim, Germany) overnight at 37 °C. To stop digestion, 1% trifluoroacetic acid (TFA) was added and the generated peptide solution was desalted using Sep-Pak tC18 cartridges (Waters, Eschborn, Germany).

Subsequently, dried peptides were dissolved in SCX Buffer A (7 mM KH_2_PO_4_, pH 2.65, 30% ACN) and separated by SCX using an Agilent 1200 HPLC equipped with a PolySULFOETHYL A column (250 × 9.4 mm; 5 μm beads, pore size 200 Å) (259-SE0502; PolyLC Inc., Columbia, SC, USA). Chromatography was performed by increasing SCX Buffer B concentration (7 mM KH_2_PO_4_, 350 mM KCl, pH 2.65, 30% ACN) from 1–30% over 40 min at a flow rate of 2 mL/min. Twelve 5-min fractions were collected over the full run, lyophilized, and subsequently desalted using Sep-Pak tC18 cartridges (Waters). Fractions 1/2, 3/4, and 11/12 were pooled for phosphopeptide enrichment, resulting in a total of 9 fractions.

### 4.4. Phosphopeptide Enrichment

A 2D affinity chromatography was conducted for phosphopeptide enrichment for each of the 9 fractions. In the first step, peptides were subjected to an immobilized metal affinity chromatography using a Ferric nitriloacatate phosphopeptide enrichment kit according to the manufacturer’s protocol (#88300; Thermo Fisher). Eluted phosphopeptides were acidified by TFA to a final concentration of 2.5% and dried by vacuum centrifugation. All flow-troughs after sample loading were pooled, dried by vacuum centrifugation, and stored for the subsequent enrichment step by metal oxide affinity chromatography using the TiO_2_ phosphopeptide enrichment spin tips (#88303; Thermo Fisher), which were used as recommended by the manufacturer. Eluted phosphopeptides were acidified with TFA to a final concentration of 1.25% and dried by vacuum centrifugation. All immobilized metal ion affinity and TiO_2_ elution fractions were cleaned up prior to MS analysis using graphite spin columns (#88302; Thermo Fisher) according to the manufacturer’s protocol.

### 4.5. Liquid Chromatography Mass Spectrometry (LC-MS)

Following phosphopeptide enrichment, dried phosphopeptides were reconstituted in 2% ACN/0.1% TFA and analyzed by an Obritrap Velos mass spectrometer connected to an Ultimate 3000 RSLC nanoflow system (Thermo Fisher). Samples were loaded on a trap column (2 cm length, 75 µm ID, 3 µm C18 particles) at a flow rate of 6 µL/min of 0.1% TFA for 5 min. The trap column was switched online with the analytical column (Acclaim PepMap, Thermo Fisher, 50 cm length, 75 µm ID, 2 µm C18 particles), and peptides were eluted at a flow rate of 250 nL/min and at 45 °C column temperature by an increasing gradient from 4 to 50% in 105 min. The column outlet was directly connected to the nano electrospray source of the mass spectrometer and peptides were ionized with a spray voltage of 1.35 kV using metal-coated fused silica emitter. 

The Orbitrap Velos mass spectrometer was operated in data-dependent acquisition mode, recording survey scans in the Orbitrap mass analyzer with a mass range from 300–1600 at a resolution of 60,000 at *m*/*z* 400. The five most intense precursors with a charge state of +2 or higher were selected for CID fragmentation with a normalized collision energy of 38, using multi-stage activation for the neutral loss masses of phosphoric acid and MS/MS spectra were acquired in the linear ion trap mass analyzer. Dynamic exclusion duration was set to 30 s.

### 4.6. Data Processing MS Data

Raw data were processed with MaxQuant software (version 1.5.3.30) [41] and peptides were identified by searching against all human entries of the UniProtKB/Swiss-Prot database (http://www.uniprot.org/) using the Andromeda search engine [42]. Propionamidation (C) was set as fixed modification, and a maximum of two missed cleavages were allowed. Phosphorylation (S/T/Y), oxidation (M), deamidation (N/Q), and acetylation (protein N-terminal) were set as variable modifications. Precursor mass tolerance was set to 5 ppm and fragment mass tolerance was set to 0.5 Da. A false discovery rate of 0.01 on peptide and protein level was used for identification, and to re-quantify and match between runs, options were checked. Common contaminants were excluded from the protein and peptide lists. For quantification, a minimum ratio count of 1 was used and peptides had to be identified in at least 3 replicates for statistical analysis. Data were analyzed and visualized with the Perseus software (version 1.5.2.6) [43,44].

### 4.7. Protein Extraction, Sodium Dodecyl Sulfate (SDS)—Polyacrylamide Gel Electrophoresis (PAGE), Western Blot Analysis, and Densitometry

Preparation of whole-cell, cytosolic, and nuclear extracts, determination of protein concentrations, electrophoresis, and Western blotting were performed as previously described [7]. For the detection of specific proteins, membranes were incubated (4 °C, overnight) with primary antibodies. Following incubation with horseradish peroxidase-coupled secondary antibodies, protein bands were visualised using enhanced chemoluminescence (ECL), SuperSignal West Femto (Thermo Fisher), or WesternBright Sirius (Advansta, Menlo Park, CA, USA) and the Bio-imaging system ECL Chemostar (Intas Science Imaging, Göttingen, Germany). For densitometric analyses, the ImageJ analysis software (National Institutes of Health, Bethesda, MD, USA) was used.

### 4.8. RNA Extraction, cDNA Synthesis, and qPCR

Cell lysis, total RNA isolation, reverse transcription of total RNA [45], and qPCR [7] were performed, as previously described. The following primers were applied: *IL8* (5′-TCCTGATTTCTGCAGCTCTGTG-3′, 5′-GGTCCACTCTCAATCACTCTC-3′), *GSK3A* (5′-ACCGCCCACTTCCCCCTCTCT-3′, 5′-GCCAGTCTGAGCTGGTCGGAG-3′), and *GSK3B* (5′-TTGCGGAGAGCTGCAAGCCG-3′, 5′-ACCCTGCCCAGGAGTTGCCA-3′). Target gene expression levels were normalized to *GAPDH* (5′-AGGTCGGAGTCAACGGAT-3′, 5′-TCCTGGAAGATGGTGATG-3′) or *β2-microglobulin* (5′-TGTGCTCGCGCTACTC-TCTCTT-3′, 5′-CGGATGGATGAAACCCAGACA-3′). Graphical representation of qPCR data was performed using GraphPad Prism 5.0 (GraphPad Software, La Jolla, CA, USA).

### 4.9. Gene Expression Data

Gene expression data of long term TNF incubated primary human monocytes were obtained from a data set published earlier [7], in which the Whole Human Genome Oligo Microarray 4x44K (Agilent, Ratingen, Germany; gene expression omnibus series accession number GSE45371) was used. 

### 4.10. Statistical/Bioinformatics Analyses

Using the GraphPad Prism 5 software, the paired two-tailed *t*-test or the one-sample *t*-test was applied, and statistical significance was defined as *p* ≤ 0.05. The analysis of the main functions of the proteins identified was performed using the data bases UniProtKD (http://www.uniprot.org/) and GeneCards (http://www.genecards.org/). Kinases binding to binding motifs enriched within the peptides with significantly increased phosphorylation were predicted applying the group-based prediction system 3.0 (http://gps.biocuckoo.org/).

## Figures and Tables

**Table 1:** Proteome: Top 25 proteins with a significantly increased level in tumor necrosis factor (TNF) long term-incubated monocytes. Using liquid chromatography and mass spectrometry (LC-MS/MS) analysis, the proteome in TNF long term-incubated and control monocytes has been analyzed. Subsequently, the ratio between the proteins in TNF long term-incubated and control cells was calculated (protein TNF/protein control). Cellular processes influenced by the respective genes are also provided.

Protein Name	Protein Ratio	Function
IDO1	19.4	metabolism
SIGLEC10	16.3	cell structure/signalling
MYH11	16	cell structure
NFKB2	15.8	gene regulation
SRSF2	10.8	gene regulation
LAD1	10.6	cell structure
CD82	9.3	cell structure/signalling
CLIC4	8.4	cell structure
KYNU	8.1	metabolism
AIM1	7.9	cell structure
FSCN1	7.6	cell structure
RELB	7	gene regulation
PPA1	7	metabolism
CYP27A1	6.5	metabolism
SET	6.1	gene regulation
SMAP	6.1	cell structure
PILRA	5.4	signalling
MPHOSPH8	5.4	gene regulation
NQO1	5.2	metabolism
IL18	5.2	signalling
TRIP10	5.1	cell structure
GRAMD1A	5.1	unknown
ADI1	5.0	metabolism
AK4	4.8	metabolism
CAST	4.6	proteolysis

**Table 2:** Phosphoproteome: Top 25 peptides with significantly increased phosphorylation in TNF long term-incubated monocytes. Using LC-MS/MS analysis, the phosphoproteome in TNF long term-incubated and naive monocytes was analyzed. Subsequently, the ratio between phosphorylated peptides in TNF long term-incubated and control cells was calculated (phosphopeptide TNF/phosphopeptide control). The affected phosphorylation sites and gene functions are also given.

Protein Name	Phosphopeptide Ratio	Phosphorylation Site	Function
EIF3G	45.9	T41	gene regulation
ITGA5	42.4	S128	cell structure
MARCKS	39.7	S101	cell structure
RBM14	35.2	S620, S623	gene regulation
VIM	29.2	S56	cell structure
MTDH	28.9	S298	cell structure
SRRM2	24.7	S2067, T2069, S2071	gene regulation
MARCKS	22.7	S77	cell structure
ADAM17	21.3	S791	proteolysis
LAD1	20.3	S38	cell structure
OGFR	19.6	S577, S637	gene regulation
SRRM2	18.6	S2067, T2069, S2071	gene regulation
FOSL2	18.2	S308	gene regulation
CD44	18.1	S697	cell structure
CD44	18.1	S706	cell structure
RELB	16.7	S573	gene regulation
NUMA1	16.7	S1757	cell structure
RFTN1	16.6	S199	cell structure/signalling
CLIP1	16.3	S195, S200	cell structure
PRRC2C	16.1	S1544	unknown
TRAF1	16.0	S66	signalling
PFKFB3	15.4	S461	metabolism
FKBP15	15.4	S1114	cell structure
LARP4B	15.4	S601	gene regulation
PPFIA1	15.0	S763	cell structure

**Table 3:** Expression and phosphorylation of proteins associated with the non-canonical nuclear factor κB (NF-κB) pathway or involved in the negative regulation of NF-κB. Expression and phosphorylation of regulatory proteins were assessed in TNF long term-incubated and naïve monocytes using microarray (mRNA; derived from analyses presented in [7]) and LC-MS/MS (protein, phosphorylation) analyses. Significantly increased ratios among TNF long term-incubated (48 h TNF) and naïve (48 h medium) cells are shown. Among the microarray and the phosphopeptide ratios, multiple values indicated different probes for one gene or different phosphopeptides of one protein (phosphoproteome).

Protein Name	Microarray Ratio	Proteome Ratio	Phosphopeptide Ratio
IDO1	55.9	19.4	-
SIGLEC10	133.3	16.3	6.2
NFKB2	4; 4.1; 4.2; 4.3	15.8	4.6
KYNU	4.8; 6.3	8.1	-
RELB	8.1	7	16.8
CAST	0.7; 0.9; 1; 1.2	4.6	13.2; 12.2; 3.4
TNFAIP2	3.1	3.75	-
C/EBPB	1.7; 1.8; 1.9; 2.9	3.4	-
TRAFD1/FLN29	4.5	3	2
USP15	0.9; 1; 1.4	2.6	-
NFKB1	1.8; 1.9; 2	2.5	-
TRAF1	6.6; 11.2	-	16
NCOR1	0.7; 1; 1.2; 1.7	-	12.9; 7.75
SIRT1	0.7	-	10.2; 5.4
PML	2.4; 2.5; 2.6; 2.9; 3.9; 6	-	8.25
DCP1A	1.4	-	7.9
NCOR2/SMRT	8.8	-	7.4; 7.4
NDRG1	4.8; 10.6	-	6.2; 4.7; 4.2; 4.0
HSF1	1	-	4.0; 4.0
TP53BP1	1.2	-	2.1

**Table 4:** Phosphoproteome: Enrichment of Gene Ontology (GO-) annotated kinase binding motifs in significantly regulated phosphopeptides identified via LC-MS/MS analysis. Kinases binding to binding motifs enriched within the peptides with significantly increased phosphorylation were analyzed using the group-based prediction system 3.0 (http://gps.biocuckoo.org/).

Gene Name	Kinase/Isoform
*ATM*	serine protein kinase ATM
*CaMK1*	Ca/calmodulin-dependent protein kinase 1
*CaMK2*	Ca/calmodulin-dependent protein kinase 2
*CDK1*	cyclin-dependent kinase 1
*CK1*	casein kinase 1
*CK2A1*	casein kinase 2α1
*CK2A2*	casein kinase 2α2
*GSK3A/B*	glycogen synthase kinase 3α/β
*MAPK11*	p38β
*MAPK12*	p38γ
*MAPK13*	p38δ
*MAPK14*	p38α
*PHKA2*	phosphorylase kinase α2
*PHKB*	phosphorylase kinase β

## Supplementary Materials

Supplementary materials can be found at https://www.mdpi.com/1422-0067/20/5/1241/s1.

## Abbreviations

ADAM	a disintegrin and metalloproteinase
ADI	arginine deiminase
AIM	apoptosis inhibitor of macrophage
AK	adenylate kinase
C/EBP	CCAAT/enhancer binding protein
CAST	Calpastatin
CD	cluster of differentiation
CLIC	chloride intracellular channel
CLIP	class II-associated invariant chain peptide
COP	constitutive photomorphogenesis
CYP	cytochrome P450
DCP	decapping mRNA protein
ECL	enhanced chemoluminescence
EIF	eukaryotic translation initiation factor
ERK	extracellular signal-regulated kinase
FCS	fetal calf serum
FGF	fibroblast growth factor
FKBP	FK506 binding protein
FOSL	Fos-related antigen
FSCN	fascin actin-bundling protein
GAPDH	glyceraldehyde-3-phosphate dehydrogenase
GRAMD	GRAM domain containing protein
GSK	glycogen synthase kinase
HDAC	histone deacetylase
HRP	horseradish peroxidase
HSF	heat shock factor
IDO	indolamin-2,3-dioxygenase
IKK	IκB kinase
IL	interleukin
IRAK	Interleukin-1 receptor-associated kinase
ITGA	Integrin alpha
IκB	inhibitor of κB
KYNU	kynureninase
LAD	ladinin
LAP	liver-enriched activating protein
LARP	La-related protein
LC	liquid chromatography
LIP	liver-enriched inhibitory protein
LPS	lipopolysaccharide
MAPK	mitogen activated protein kinase
MARCKS	myristoylated alanine-rich C-kinase substrate
MEM	minimum essential medium
miR	mircoRNA
MPHOSPH	M-phase phosphoprotein
MS	mass spectrometry
MTDH	metadherin
MYH	myosin heavy chain
NCOR	nuclear receptor co-repressor
NDRG	N-myc downregulated gene
NF-κB	nuclear factor κB
NQO	NAD(P)H Quinone Dehydrogenase
NUMA	nuclear mitotic apparatus protein
OGFR	opioid growth factor receptor
OPI	oxaloacetate/pyruvate/insulin
PAGE	polyacrylamide gel electrophoresis
PDK	phosphoinositide-dependent kinase
PFKFB	6-phosphofructo-2-kinase/fructose-2,6-biphosphatase
PILRA	paired immunoglobin-like type 2 receptor α
PML	promyelocytic leukemia protein
PMSF	phenylmethylsulfonyl fluoride
PPA	pyrophosphatase
PPFIA	protein tyrosine phosphatase receptor Type F interacting protein
PRRC	proline-rich and coiled-coil-containing protein
RBM	RNA binding motif protein
REL	v-rel reticuloendotheliosis viral oncogene homolog
RFTN	Raftlin
RPMI	Roswell Park Memorial Institute
SDS	sodium dodecyl sulfate
SET	SET nuclear proto-oncogene
SHP	Src homology region 2 domain-containing phosphatase
SIGLEC	Sialic acid-binding immunoglobulin-type lectin
SIRT	sirtuin
SMAD	SMAD family member
SMAP	small acidic protein
SRRM	serine/arginine repetitive matrix protein
SRSF	serine/arginine-rich splicing factor
TBP	TATA-binding protein
TGF	transforming growth factor
THP-1	Tohoku Hospital Pediatrics-1
TNF	tumor necrosis factor
TNFAIP	tumor necrosis factor α-induced protein
TP53BP	tumor suppressor p53-binding protein
TRAF	TNF receptor-associated factor
TRAFD	TRAF-type zinc finger domain-containing protein
TRIP	thyroid receptor-interacting protein
U	unit
USP	ubiquitin specific peptidase
VIM	vimentin
